# Cognitive Function Assessment Using a Virtual Reality Serious Game System in Patients With Stable Schizophrenia: Prospective Cohort Study

**DOI:** 10.2196/83001

**Published:** 2026-05-08

**Authors:** Xingxing Li, Yu Zhuo, Xiandong Meng, Wenting Zhao, Chenxin Wu, Kai Yan, Leiyu Yue, Yu Sun, Qian Xiong, Xi Cao, Xiaomin Kou, Jianying Yu

**Affiliations:** 1 Mental Health Center, National Center for Mental Disorders West China Hospital, West China School of Nursing Sichuan University Chengdu, Sichuan China; 2 Department of Maternal, Child and Adolescent Health West China School of Public Health and West China Fourth Hospital Sichuan University Chengdu, Sichuan China

**Keywords:** B-CATS, cognitive function, schizophrenia, serious game, virtual reality

## Abstract

**Background:**

Cognitive impairment is a core and enduring deficit in schizophrenia, severely affecting social functioning and quality of life. Traditional assessments such as the MATRICS Consensus Cognitive Battery face limitations in validity and engagement. Virtual reality (VR) serious games may offer an immersive alternative, and machine learning (ML) can uncover complex behavioral patterns. However, integrating VR-based assessment with ML for discriminating stable-phase schizophrenia remains unexplored.

**Objective:**

This prospective cohort study aimed to examine whether a VR serious game (“Fruit Pioneer”) can effectively assess cognitive function in stable schizophrenia, verify its correlation with the standard Brief Cognitive Assessment Tool for Schizophrenia (B-CATS), and test the discriminative capacity using ML models. We hypothesize that (1) patients with schizophrenia will show poorer VR game performance than healthy controls (HCs), (2) VR metrics will correlate with B-CATS scores, and (3) ML models will help classify patients with schizophrenia and HCs using VR data.

**Methods:**

A total of 42 patients with stable schizophrenia and 65 HCs (aged 18-40 years) were enrolled. Exclusion criteria included color blindness, visual impairment, substance abuse, and comorbid chronic physical diseases. Finally, 39 patients with schizophrenia and 64 HCs were included. Materials included the VR serious game “Fruit Pioneer,” B-CATS (Digital Symbol Substitution Test, Trail Making Test Part A, Trail Making Test Part B, and Animal Fluency), Simulator Sickness Questionnaire, and Game Experience Questionnaire. Data were collected via standardized VR gameplay and paper-based assessments. Logistic regression and a support vector machine (SVM) model were built using VR metrics.

**Results:**

Patients with schizophrenia performed worse on all B-CATS subtests (all *P*<.001). They also showed lower VR total scores (median 467, IQR 376-544 vs median 683, IQR 616-753; *P*<.001), longer reaction times (median 1.11, IQR 0.995-1.23 vs median 1.03, IQR 0.96-1.1; *P*=.006), lower gaze hit rates (median 0.515, IQR 0.442-0.554 vs median 0.552, IQR 0.497-0.592; *P*=.01), and higher bomb penalty scores (median 150, IQR 95-170 vs median 108, IQR 85-131; *P*=.002). In the schizophrenia group, VR metrics correlated with B-CATS results, whereas this relationship was minimal in HCs. Classification performance of the SVM (average area under the curve [AUC]=0.874, 95% CI 0.860-0.888) was comparable to logistic regression (average AUC=0.854, 95% CI 0.838-0.870).

**Conclusions:**

This study demonstrates the innovative integration of a VR serious game with ML to assess cognitive function in stable schizophrenia. Unlike prior VR studies focused mainly on validation, our approach combines behavioral metrics with an SVM model, achieving effective classification. The findings support the potential of a scalable digital assessment correlated with standard tests. In clinical practice, this system may serve as an engaging alternative to traditional methods, facilitating long-term cognitive monitoring and personalized rehabilitation strategies.

## Introduction

### Problem

Schizophrenia imposes significant economic and psychological burdens on patients, their families, and society [1]. Cognitive dysfunction, a core feature affecting the majority of patients, impairs attention, memory, executive function, and processing speed [2,3], thereby severely compromising social functioning and quality of life. This underscores the critical need for accurate assessment tools. Traditional cognitive assessments, for example, the MATRICS Consensus Cognitive Battery, however, face major limitations [4]. They are time-intensive and require specialized training, leading to low clinical use; their artificial tasks lack validity and fail to reflect real-world functioning. Additionally, they are prone to subjectivity (unable to capture fine-grained behavioral metrics), and their passive nature often results in low patient motivation and compliance [5,6]. The Brief Cognitive Assessment Tool for Schizophrenia (B-CATS) improves time-efficiency but remains a laboratory-based assessment [7]. While it evaluates key domains such as processing speed, attention, executive function, and semantic memory, it still lacks validity and the capacity for dynamic behavioral capture.

### Review of Relevant Scholarship

Emerging technologies such as virtual reality (VR) and serious games offer potential solutions. VR creates immersive and standardized environments that can enhance engagement through reward mechanisms, which is particularly crucial for patients with anhedonia [8-10]. Furthermore, it enables the automated, objective capture of high-resolution behavioral data, such as gaze latency and movement precision [11]. Empirical studies support the use of VR in effectively assessing and differentiating cognitive impairments in psychiatric populations. A VR navigation task study demonstrated that patients with schizophrenia exhibited significant impairments in allocentric, egocentric, visual, and verbal memory tasks compared to bipolar disorder patients and healthy controls (HCs) [12]. Another study used interactive VR to assess verbal memory, processing speed, attention, working memory, and planning skills in 40 patients with mood or psychotic disorders [13]. Serious games, which are being increasingly adopted in mental health research and clinical practice, integrate cognitive assessment into structured virtual activities and foster engagement that aligns with core therapeutic goals [14].

Considering VR assessments generate complex and high-dimensional data, machine learning (ML) has been recommended as a core analytical component [15,16]. Support vector machines (SVMs), as a famous ML model, can identify subtle, nonlinear patterns and support individualized cognitive profiling for this heterogeneous disorder [17]. Compared with the traditional logistic regression analysis, SVM handles high-dimensional data well and mitigates overfitting with limited samples [18]. Recent studies have also validated that SVM models using VR data can achieve superior classification accuracy [19].

### Hypothesis, Aims, and Objectives

This study adapts our previously developed VR serious game “Fruit Pioneer” [20], aiming to investigate the efficacy of this serious game system in assessing cognitive dysfunction in patients with stable schizophrenia. Specifically, it seeks to evaluate the system’s validity through correlation analysis with the B-CATS and to leverage ML to validate its discriminative capacity in differentiating patients from HCs. By integrating the validity of VR, the engagement of serious games, and the pattern recognition capabilities of ML, this study may provide a scalable and clinically relevant cognitive assessment tool. We hypothesize that (1) patients will exhibit poorer overall performance on the VR serious game compared to HCs; (2) metrics from the VR serious game will show potential correlations with B-CATS scores in the patient group; and (3) ML models will classify patients and HCs based on the VR game metrics.

## Methods

### Inclusion and Exclusion

This study was a prospective cohort study conducted in patients with stable schizophrenia and HCs at the Mental Health Center of West China Hospital, Sichuan University, from May to December 2023. Participants were diagnosed with stable-phase schizophrenia according to the *Diagnostic and Statistical Manual of Mental Disorders,* *Fifth Edition* (*DSM-5*). The stable phase was defined as a period satisfying two key criteria: (1) no acute exacerbation of positive symptoms within the past 3 months and (2) maintenance on a stable dosage of antipsychotic medication for at least 1 month. Inclusion criteria for the schizophrenia group included (1) age 18-40 years, (2) inpatients in the stable phase of schizophrenia, and (3) signed written informed consent. Exclusion criteria for the schizophrenia group included (1) color blindness, (2) visual impairment requiring glasses for correction, (3) substance abuse (including alcohol dependence), and (4) comorbid chronic physical diseases. Inclusion criteria for the HCs group were (1) age 18-40 years, (2) healthy individuals with no personal or family history of psychiatric disorders (confirmed via self-report and brief structured interview), and (3) signed written informed consent. Exclusion criteria for HCs were consistent with the schizophrenia group.

### “Fruit Pioneer” Serious Game Procedures

We have developed the VR serious game “Fruit Pioneer” [20], aiming to provide an immersive, distraction-minimized environment to enhance assessment engagement. The game adopts a first-person perspective, featuring key elements including a swinging panda, a real-time scoreboard, and multiple circular fruit-shooting nozzles (Figure S1 in Multimedia Appendix 1). To minimize external distractions, participants wore VR headsets and earphones that delivered dynamic game sounds (eg, fruit-slicing feedback and background music). Handheld interactive devices were visualized as 2 long swords in the virtual environment, enabling intuitive operation.

To eliminate potential learning effects that may confound cognitive performance outcomes, no practice session was conducted prior to the formal assessment; instead, trained researchers provided participants with a standardized verbal explanation of the game rules, including fruit/bomb identification, scoring criteria, controller operation, and movement boundaries within the designated physical area. Following the rule explanation, the formal 5-minute game session was initiated, during which fruits (banana, apple, watermelon, orange, and pineapple) and black spherical bombs were randomly launched from the 8 nozzles at varying speeds (Figure S1 in Multimedia Appendix 1), and participants could move their head and body freely within the designated physical area to adjust their field of view and swing the haptic controllers to slice fruits for points while avoiding bombs.

A predefined scoring system has been implemented in this framework to distinguish cognitive task difficulty. Specifically, watermelon (+1 point/piece) has the largest volume, highest visual salience, and lowest difficulty, requiring minimal attentional allocation for perception and slicing; banana (+2 points/piece) has moderate volume and irregular shape, with intermediate visual salience and difficulty that demands moderate attentional effort for tracking and slicing; apple, orange, and pineapple (+3 points/piece each) have small volume and similar round shapes, resulting in the lowest visual salience and highest difficulty, and their low salience and similar appearance increase perceptual challenge, requiring participants to strategically allocate attention and prioritize low-salience targets—tasks relying on executive function and sustained attention, which are impaired in schizophrenia. Bombs served as penalty targets (–5 points/piece) to assess inhibitory control (a core component of executive function), as the design requires participants to suppress prepotent slicing responses to task-irrelevant stimuli (bombs). The game automatically terminated after 5 minutes, with all behavioral data recorded in real time by the system, and a professional psychiatric nurse was present throughout the gameplay to guide participants, ensure the smooth execution of the assessment, and promptly address any adverse events (eg, anxiety and dizziness).

A total of 9 core metrics were extracted from the game to assess participants’ cognitive functions:

1. Game score: reflects overall operation performance, including points from all sliced fruits and deductions from sliced bombs; the total score is the sum of all items. Formula: total game score = Σ(score of each sliced fruit) – Σ(penalty score of each sliced bomb). Watermelon score = quantity of sliced watermelons × (+1 point/piece) Banana score = quantity of sliced bananas × (+2 points/piece) Apple score = quantity of sliced apples × (+3 points/piece) Orange score = quantity of sliced oranges × (+3 points/piece) Pineapple score = quantity of sliced pineapples × (+3 points/piece) Bomb penalty score = quantity of sliced bombs × (+5 points/piece)
2. Average reaction time for hits‌: measures the mean duration from target (fruit/bomb) appearance to successful interaction, reflecting integrated attentional and executive function capabilities involving working memory processing efficiency. Formula: average reaction time for hits = total reaction time of all successful hits/number of successful hits; reaction time for a single hit = time of target appearance (system record) – time of successful slicing (system record).
3. ‌Average time from gaze to hit: captures the mean latency between visual fixation on a target and execution of the corresponding action, directly associated with decision-making speed and cognitive control within executive functions. Formula: average time from gaze to hit = total time from gaze to hit of all successful hits/number of successful hits; time from gaze to hit = time of successful slicing – time of initial visual fixation on the target (tracked by VR eye-tracking module).
4. Gaze hit rate‌: represents the proportion of successful operations under visual fixation, quantifying the accuracy of goal-directed behavior and the efficacy of executive responses. Formula: gaze hit rate = (number of successful hits with prior visual fixation/total number of targets fixated on) × 100%.
5. Nongaze hit rate‌: indicates the proportion of successful operations without prior visual fixation, supplementing the assessment of automated and intuitive executive responses. Formula: nongaze hit rate = (number of successful hits without prior visual fixation/total number of targets fixated on) × 100%.
6. Shortest time from gaze to hit‌: identifies the fastest response latency, signaling peak performance of attention and executive function under optimal conditions. Formula: shortest time from gaze to hit = minimum value among the time from gaze to hit of all successful hits.
7. Longest time from gaze to hit‌: reveals the slowest response latency, exposing potential deficits in cognitive flexibility or task-switching efficiency. Formula: longest time from gaze to hit = maximum value among the time from gaze to hit of all successful hits.
8. Bomb score‌: quantifies inhibitory control—a core component of executive function—by measuring suppression of responses to irrelevant stimuli. Formula: bomb score = number of bombs hit × penalty score per bomb. Note: penalty score per bomb is predefined as 5 points; a higher bomb score indicates poorer inhibitory control, as it reflects more frequent inappropriate responses to irrelevant stimuli (bombs).
9. Number of continuous hits‌: tracks consecutive successful operations, reflecting sustained attention, working memory updating, and executive coordination. Formula: number of continuous hits = number of consecutive successful fruit slicing operations (even 2 consecutive successful slicing operations are counted as valid continuous hits). Note: “Continuous” is defined as successful fruit slicing without interruptions (slicing bombs or missing fruits), and this continuous slicing process is automatically recorded by the system.

### Cognitive Assessment by B-CATS

B-CATS is used in this study for objective cognitive function assessment across 4 dimensions: Digital Symbol Substitution Test (DSST), Trail Making Test Part A (TMTA), Trail Making Test Part B (TMTB), and Animal Fluency (AF; Table S4 in Multimedia Appendix 1) [21]. In the DSST, participants received a sheet displaying 9 symbols, each matched with digits 1-9 at the top, with additional rows of symbols below. Within a 120-second time frame, participants were required to correctly match each symbol to its respective digit. The total number of correct matches determined the score. Out of 120 symbols on the sheet, the first 10 served as examples, so the highest possible score was 110. The DSST quantitatively assessed participants’ core cognitive domains, including ‌processing speed, attentional control, and visual working memory‌‌. For TMTA, participants sequentially connected numbered circles (1-2-3-...) in ascending order while maintaining continuous pen contact. Errors were corrected by redirecting to the last correct position. TMTA assesses visual scanning speed, sustained attention, and psychomotor processing speed. For TMTB, participants alternately connected numbered and lettered circles (1-A-2-B-3-...) in ascending alphanumeric sequence under continuous pen-tracking requirements, with errors similarly corrected. TMTB evaluates executive function, task-switching ability, and inhibitory control (specifically requiring suppression of prepotent numeric sequencing). ‌In the AF task, participants generated unique animal names within a 60-second interval, with the score representing the total count of valid distinct responses. AF assesses three core domains: (1) semantic memory (access to categorical knowledge mediated by anterior temporal lobes), (2) lexical access efficiency (rapid word retrieval supported by left inferior frontal gyrus), and (3) strategic retrieval processes (executive-guided clustering/switching strategies involving dorsolateral prefrontal cortex). Performance thus reflects integrated frontotemporal network functions with significant executive control contributions.

### Simulator Sickness Questionnaire

The Simulator Sickness Questionnaire (SSQ), a widely-used tool in VR research, evaluates discomfort symptoms that participants may experience post engagement with VR environments (Table S5 in Multimedia Appendix 1). The SSQ assesses 16 different symptoms across 3 dimensions: oculomotor issues, disorientation, and nausea. In our study, the scoring procedure for the SSQ followed the recommendations by Bouchard et al [22]: the total nonweighted score (ie, SSQ-total raw) was calculated by simply summing all 16 items of the SSQ once [23].

### Game Experience Questionnaire Core Module

The Game Experience Questionnaire Core Module (GEQ-Core Module), designed by IJsselsteijn et al [24], encompasses essential aspects necessary for assessing the gaming experience (Table S6 in Multimedia Appendix 1) [25]. It includes 33 items across 7 dimensions: competence, sensory and imaginative immersion, flow, challenge, positive affect, negative affect, and tension/annoyance. The questionnaire uses a 5-point Likert scale for responses, ranging from 0 (not at all) to 1 (slightly), 2 (moderately),3 (highly), and 4 (extremely). By calculating the average scores for each dimension, differences in participants’ experiences across various aspects can be evaluated.

### Analytic Strategy

A total of 103 participants were included for the final analyses, and no missing data existed. All analyses were performed according to a prespecified statistical analysis plan. Statistical comparisons used a 2-tailed significance test with a 95% confidence level. All analyses were conducted using a complete case analysis. All statistical analyses were performed using R software (version 4.2.3; R Foundation for Statistical Computing).

For continuous variables (eg, B-CATS subtest scores, VR game metrics, SSQ, and GEQ-Core Module scores), the Shapiro-Wilk test was first used to verify data normality: if the data were normally distributed, the independent samples 2-tailed *t* test was used for intergroup comparisons; if not normally distributed, the Mann-Whitney *U* test (nonparametric test) was applied. The chi-square test was used for statistical inference of categorical data (eg, gender distribution between groups).

**Figure 1:**
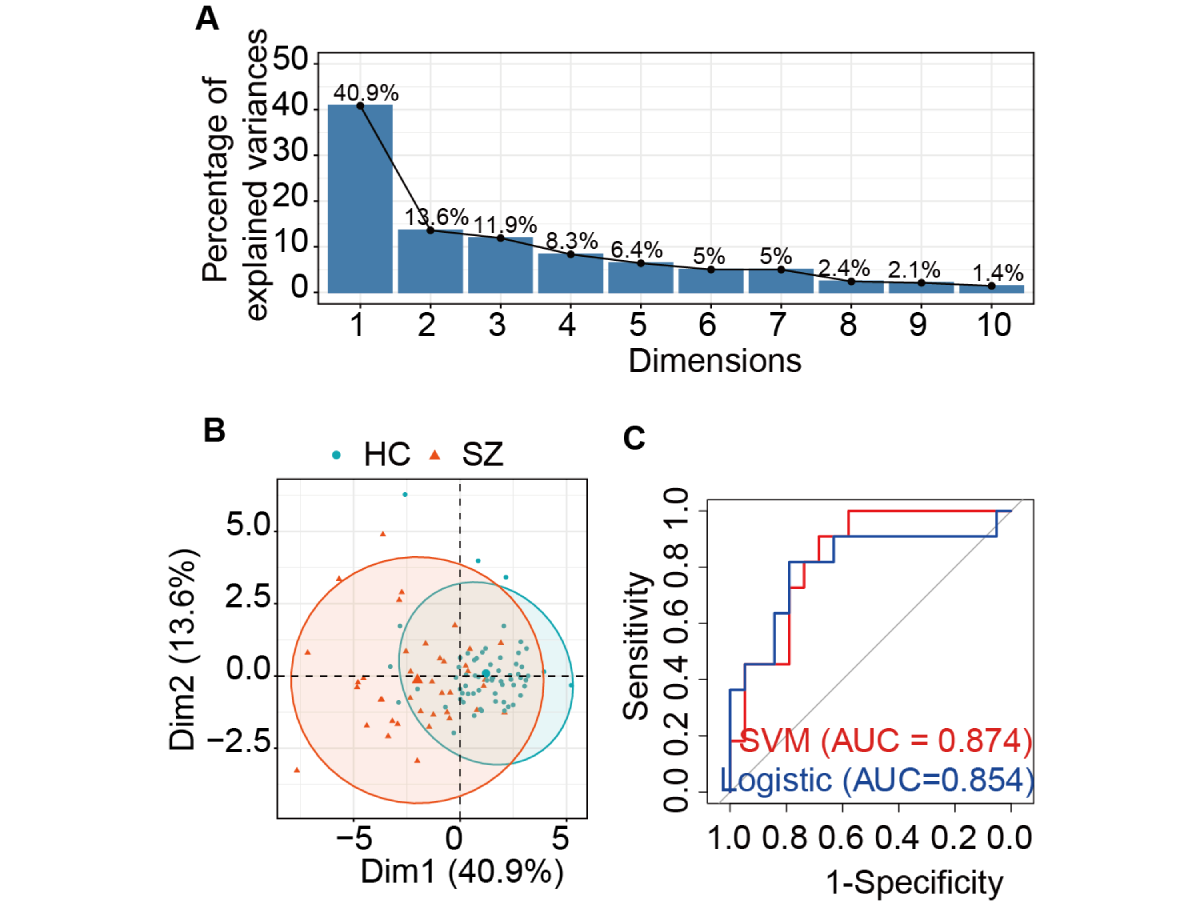
Principal component analysis and receiver operating characteristic curves for support vector machine (SVM) and logistic regression machine models. (A) Scree plot of principal component analysis. (B) Principal component analysis score plot of samples. (C) Receiver operating characteristic curves for classification models. AUC: area under the curve.

To identify the most discriminative VR metrics for classifying patients with schizophrenia and HCs, we performed principal component analysis (PCA). All 15 standardized VR game metrics (game score, banana score, apple score, watermelon score, orange score, pineapple score, bomb score, average reaction time for hits, average time from gaze to hit, gaze hit rate, nongaze hit rate, shortest time from gaze to hit, longest time from gaze to hit, bomb penalty score, and number of continuous hits) were included in the PCA. This technique transforms the correlated original variables into a set of uncorrelated principal components (PCs), which are ordered by the amount of variance they explain. To determine the number of key PCs to retain for subsequent analysis, we examined the scree plot (Figure 1A) and the cumulative proportion of explained variance. The scree plot showed a clear “elbow” after the first few components, indicating a point of diminishing returns in variance explained by subsequent components. We retained the first 7 PCs (“‌nongaze hit rate,” “‌gaze hit rate,” “orange score” “apple score,” “pineapple score,” “watermelon score,” and “bomb score”), which collectively explained over 80% of the total variance in the dataset, ensuring a substantial reduction in dimensionality while preserving most of the original information. To address potential bias from unbalanced group distribution or data leakage, we used stratified random sampling (via the createDataPartition function in the caret package) to split the full dataset into a 70% training set and 30% testing set in each iteration (total 50 iterations). For model training, a radial kernel SVM was trained on the training set with probability estimates enabled to generate continuous prediction scores for receiver operating characteristic analysis, and a binomial logistic regression model was trained on the same training set to predict binary group membership. For performance evaluation and robustness check, model performance was evaluated on the independent testing set for each iteration using accuracy, precision, recall (sensitivity), specificity, and *F*_1_-score. Receiver operating characteristic curves and area under the curve (AUC) were calculated to quantify discriminative ability. The entire process was repeated 50 times with independent stratified random splits to account for random variation, mean values of all metrics (±variability) were reported to reflect stable model performance, and 90% CIs for AUC values were additionally calculated to assess the reliability of discriminative ability estimates.

### Ethical Considerations

This study was conducted in strict accordance with the ethical principles of the Declaration of Helsinki and approved by the Medical Research Ethics Committee of Sichuan University (reference number 2023-1926). Safety was ensured by an on-site psychiatric nurse monitoring for adverse events (eg, anxiety and dizziness) with authority to terminate assessments if needed.

All participants provided written informed consent after detailed verbal explanations of study objectives, procedures, potential risks, benefits, and the right to withdraw without penalty. HCs received additional clarification about voluntary participation. Original consent forms are securely archived. Ethics approval explicitly permits future secondary data analysis without reconsent. All data were anonymized. Personally identifiable information was stored separately in password-encrypted systems with restricted access; paper records were kept in locked cabinets. Only authorized researchers accessed deidentified data. No identifiable information appears in publications. Data retention complies with institutional regulations. No form of compensation was provided to participants for their participation in this study. This publication and its supplementary materials do not contain any images, videos, or other materials that may identify individual participants.

## Results

### Participant Flow and Recruitment

**Figure 2:**
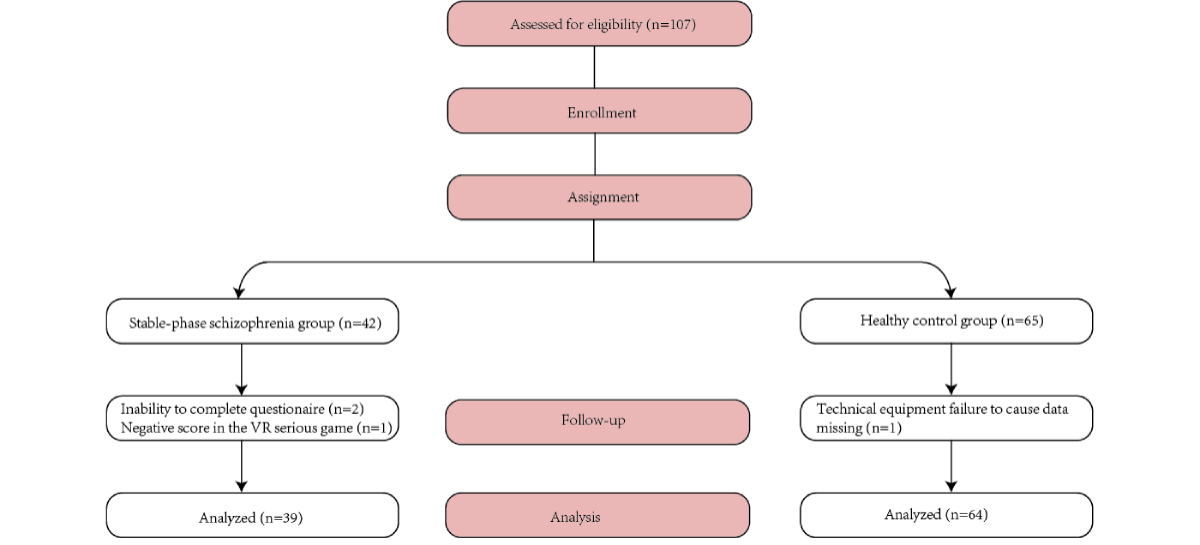
Flowchart of the data selection process with reasons for exclusion of participants specified. In the stable-phase schizophrenia group, 3 participants were excluded from the final analysis: 2 were unable to complete the questionnaire, and 1 had a negative score in the virtual reality (VR) serious game, resulting in 39 participants analyzed. In the HC group, 1 participant was excluded due to technical equipment failure that caused data to be missing, leaving 64 participants for final analysis.

A total of 107 participants were initially enrolled, consisting of 42 patients with schizophrenia and 65 HCs. Overall, 103/107 (96.26%) participants completed the study. For the schizophrenia group, 3 out of the 42 initially enrolled patients were excluded: 2 due to inability to complete TMTB (unfamiliar with English alphabetical order) and 1 due to a negative total score in the VR serious game (indicating misunderstanding of game rules). For the HCs group, 1 out of the 65 initially enrolled controls was excluded due to technical equipment failure that prevented data recording. After applying post–data-collection exclusion criteria, 39 patients with schizophrenia and 64 HCs were included in the final analytic sample. No missing data were observed in the final analytic sample (Figure 2).

The schizophrenia group was older than the HCs group (patients with schizophrenia: median 25, IQR 19-29 years; HCs: median 20, IQR 20-21 years; *P*<.001). No significant between-group differences were observed in gender distribution (*χ*^2^_1_=0.072; *P*=.79) or educational background (*χ*^2^_1_=1.196; *P*=.27), indicating that the groups were balanced in these demographic variables (Table 1).

**Table 1:** Demographic characteristics of stable patients with schizophrenia (SZs) and healthy controls (HCs) in a cross-sectional study of virtual reality serious game–based cognitive function assessment.

Group		All	HCs	SZs	Test statistic		*P* value	
**Sex, n**						0.072^a^ (1)		.79
	Male	40	26	14				
	Female	63	38	25				
Age (years), median (IQR)		20 (20-23)	20 (20-21)	25 (19-29)	737^b^ (101)		<.001	
**Education background, n**						1.196^a^ (1)		.27
	Bachelor's or below	101	64	37				
	Bachelor's or above	2	0	2				

^a^Chi-square test.

^b^Mann-Whitney *U* test.

### B-CATS Outcomes of Patients With Schizophrenia and HCs

The DSST scores and AF scores followed a normal distribution (*P*>.05), while the TMTA and TMTB scores did not conform to a normal distribution (*P*<.01). Therefore, with age as a covariate, an independent *t* test was used to compare the DSST and AF scores, and the Mann-Whitney *U* test was used to compare the TMTA and TMTB scores between the 2 groups. Significant differences were observed between patients with schizophrenia and HCs across B-CATS results. The schizophrenia group exhibited lower performance on DSST (*P*<.001), TMTA (*P*<.001), TMTB (*P*<.001), and AF (*P*<.001) compared to the HCs group (Table 2).

**Table 2:** Comparison of baseline cognitive function (assessed via the Brief Cognitive Assessment Tool for Schizophrenia) between patients with schizophrenia (SZs) and healthy controls (HCs) in a cross-sectional study.

Variables	HCs	SZs	Test statistic	*P* value
DSST^a^, mean (SD)	65 (9.30)	37.3 (10.8)	13.136^b^ (101)	<.001
TMTA^c^, median (IQR)	22 (19-26.2)	55 (41.5-77)	87^d^ (101)	<.001
TMTB^e^, median (IQR)	45.5 (35-55.5)	132 (88-216)	141^d^ (101)	<.001
AF^f^, mean (SD)	25.4 (6.07)	16.1 (5.73)	7.875^b^ (101)	<.001

^a^DSST: Digital Symbol Substitution Test.

^b^*t* test.

^c^TMTA: Trail Making Test Part A.

^d^Mann-Whitney *U* test.

^e^TMTB: Trail Making Test Part B.

^f^AF: Animal Fluency.

### Performance of VR Serious Game Between Patients With Schizophrenia and HCs

Patients with schizophrenia achieved lower scores for various fruits but higher penalty points for bombs in the VR serious game compared to HCs (*P*<.001; Table 3). Furthermore, patients with schizophrenia exhibited longer average reaction time for hits (*P*<.001) and average time from gaze to hit (*P*<.001) compared to HCs. Also, patients with schizophrenia had a lower gaze hit rate compared to HCs (*P*=.01). However, no discernible differences were observed between patients with schizophrenia and HCs in terms of nongaze hit rate, nongaze/gaze hit rate, shortest time from gaze to hit, longest time from gaze to hit, and the number of continuous hits. In the schizophrenia group, significant correlations were observed between the B-CATS and total game score (*P*=.04), ‌average reaction time for hits (*P*=.02), continuous hits (*P*=.03), as well as shortest time from gaze to hit (*P*=.02; Table S2 in Multimedia Appendix 1).

**Table 3:** Comparison of virtual reality serious game performance between patients with schizophrenia (SZs) and healthy controls (HCs) in a cross-sectional study.

Variables			HCs		SZs		Test statistic		*P* value
**Game score (points)**									
	Total score, median (IQR)	683 (616-753)		467 (376-544)		2255.5^a^ (101)		<.001	
	Banana score, mean (SD)	70.7 (11.5)		53.0 (13.6)		6.766^b^ (101)		<.001	
	Apple score, mean (SD)	197 (27.8)		150 (35.8)		7.013^b^ (101)		<.001	
	Watermelon score, median (IQR)	102 (93.8-107)		84 (76-93.5)		2137.5^a^ (101)		<.001	
	Orange score, mean (SD)	182 (29.1)		138 (34.5)		6.625^b^ (101)		<.001	
	Pineapple score, mean (SD)	240 (36.3)		180 (40.8)		7.596^b^ (101)		<.001	
	‌Bomb penalty score, median (IQR)	108 (85-131)		150 (95-170)		797.5^a^ (101)		.002	
Average reaction time for hits (seconds), median (IQR)			1.03 (0.96-1.1)		1.11 (0.995-1.23)		845.5^a^ (101)		.006
Average time from gaze to hit (seconds), median (IQR)			0.43 (0.389-0.468)		0.481 (0.417-0.599)		807.5^a^ (101)		.003
Continuous hits, median (IQR)			9.34 (7-12)		10 (7-12)		1227.5^a^ (101)		.89
Gaze hit rate, median (IQR)			0.552 (0.497-0.592)		0.515 (0.442-0.554)		1626^a^ (101)		.01
Nongaze hit rate, median (IQR)			0.394 (0.337-0.434)		0.402 (0.326-0.45)		1211^a^ (101)		.80
Shortest time from gaze to hit (seconds), median (IQR)			0.007 (0.006-0.007)		0.007 (0.007-0.007)		1087.5^a^ (101)		.21
Longest time from gaze to hit (seconds), median (IQR)			3.87 (2.59-5.35)		3.90 (2.57-6.89)		1197.5^a^ (101)		.73

^a^Mann-Whitney *U* test.

^b^*t* test.

### Results of SSQ and GEQ-Core Module

While no statistically significant differences were found between the patients with schizophrenia and HCs in the dimensions of competence, immersion, flow, and challenge, notable distinctions emerged between the symptom group and the HC group in their scores for positive and negative affect, as well as tension (Table S3 in Multimedia Appendix 1). The HCs group scored higher in positive affect, and the schizophrenia group scored higher in negative affect (*P*=.03 and *P*=.02, respectively). Tension was significantly higher in the schizophrenia group compared to the HCs group (*P*=.045). Regarding SSQ scores, although the difference between the schizophrenia group and the HCs group did not reach the conventional threshold for statistical significance (*P*=.06), this value represents a borderline trend that merits cautious interpretation. Specifically, the numerical pattern of SSQ scores suggested a potential directional difference between the 2 groups, which may have been masked by the relatively small sample size of this study.

### ML Model Classification Results

Based on PCA analysis, 7 features were included in ML analyses, including “‌nongaze hit rate,” “‌gaze hit rate,” “orange score,” “apple score,” “pineapple score,” “watermelon score,” and “bomb score”. SVM had a slightly higher AUC value compared with logistic regression machine (LRM) models (AUC—SVM: 0.874, 95% CI 0.860-0.889; LRM: 0.854, 95% CI 0.838-0.870; Figure 1). Both SVM and LRM models effectively classified patients with schizophrenia and HCs, with SVM achieving 0.801 accuracy, 0.765 precision, 0.710 *F*_1_-score, and LRM showing 0.794 accuracy, 0.734 precision, 717 *F*_1_-score.

## Discussion

### Principal Findings

Our study demonstrated that the VR serious game could differentiate cognitive function between patients with schizophrenia and HCs. Furthermore, VR-derived metrics showed potential correlations with B-CATS measures, highlighting the potential use of VR-based serious games for capturing valid cognitive functioning.

### Interpretation

Patients with schizophrenia exhibit significant cognitive impairment, including attention deficits and executive function deficits. Attention deficits manifest as difficulties in sustaining attention on task objectives, particularly evident in multitasking contexts [26]. According to stimulus-driven theories of visual processing within the current VR serious game, external stimuli can directly generate perceptual experiences corresponding to the stimulus properties [27]. Constrained by the physiological limitations of the visual system, the capacity for processing external stimuli at any given moment is finite [28]. This limitation manifests in the domain of stimulus size, where larger objects inherently exert stronger visual stimulation intensity compared to smaller objects [29]. Within the VR serious game, when multiple fruits appear simultaneously, larger fruits deliver higher visual stimulus intensity, while smaller fruits deliver lower visual stimulus intensity [20]. Therefore, smaller fruits are assigned higher point values, while larger fruits receive lower scores in the current VR games. This score system would help distinguish patients with attention deficits. This aligns with their poorer performance evaluated by B-CATS. Compared to HCs, patients with schizophrenia also exhibited prolonged response latencies across VR tasks, which correspond to impairments observed in the DSST and TMT subtests of the B-CATS.

Inhibitory control constitutes a core component of executive function [30], primarily serving to suppress prepotent responses [31]. Critically, patients with schizophrenia experiencing heightened excitement face an elevated risk of impulsive/aggressive behaviors, posing potential threats to the environment, themselves, and others [32]. During the VR game, these patients may exhibit undirected behavioral actions, consequently resulting in poor game performance [20]. Furthermore, these behaviors may precipitate hyperarousal and elevated mood states [31]. Therefore, within this VR game framework, bombs were incorporated as inhibitory stimuli. Accidental detonation of these bombs triggers deduction of accumulated points according to a predefined scoring protocol, thereby operationalizing the inhibitory mechanism [20]. This finding aligns with impaired inhibition measured by the TMT-B, wherein suppressing prepotent numerical sequencing tendencies is essential.

### Similarity of Results

Patients with schizophrenia exhibited lower scores in positive emotions and higher scores in negative emotions and tension in our study, aligning with prior research indicating a tendency for individuals with schizophrenia to anticipate negative emotions more than HCs [33,34]. Martin et al [35] conducted a study involving 16 patients with schizophrenia and 30 HCs using a task paradigm with real-life social interaction, which similarly found heightened anticipation of negative emotions among patients with schizophrenia compared to HCs. Furthermore, there were no differences in SSQ scores between the schizophrenia group and the HCs group, indicating comparable susceptibility to simulator sickness symptoms between patients with schizophrenia and HCs. This implies that patients with schizophrenia's adaptability in VR environments may mirror that of nonclinical populations.

Our current VR serious game helped differentiate the patients with schizophrenia from the HCs using ML. This aligned with a previous study on VR assessment of cognitive function in patients with schizophrenia [36]. The authors stated that the VR cognitive training system distinguished patients with schizophrenia in the stable phase stage and HCs with high accuracy (88.89%), sensitivity (88.89%), and specificity (88.89%) [36]. While the classification accuracy of SVM models remained moderate in the study, this limitation does not negate the core value of ML integration. Rather than evaluating ML by accuracy alone, its indispensable contribution lies in 3 key aspects that address the shortcomings of traditional analytical methods. First, SVM excels at processing high-dimensional, multimodal VR-derived data (eg, gaze latency and movement precision) that the traditional LRM method cannot fully interpret. Conventional approaches only detect linear relationships between variables, but ML identifies hidden nonlinear interactions and prioritizes key cognitive markers (eg, bomb score for inhibitory control deficits). Second, from a clinical perspective, SVM provides individualized cognitive profiles that the traditional B-CATS tool cannot deliver. Even with moderate accuracy, the models generate targeted deficit profiles for each patient, guiding personalized rehabilitation planning. Third, this limited performance reflects objective constraints rather than SVM ineffectiveness, including the moderate sample size, inherent heterogeneity of schizophrenia, and incomplete coverage of cognitive domains by existing VR metrics.

### Implications

This study provides preliminary evidence supporting the potential use of VR serious games as an adjunctive tool for cognitive assessment in stable schizophrenia. Our findings suggest that the “Fruit Pioneer” game might address key limitations of traditional testing by offering a more engaging format that captures nuanced behavioral data. This may, in future applications, improve patient acceptance and facilitate more frequent monitoring in clinical or research settings. Methodologically, this work contributes a scalable digital assessment framework. The integration of game mechanics with ML for pattern recognition represents a promising step toward automated, objective cognitive profiling. However, the translational clinical implications remain to be fully established. Future work must prioritize independent validation in larger, age-matched cohorts and longitudinal designs to determine the tool’s reliability and sensitivity to change.

### Limitations

This study has several limitations. First, the ML models were developed and evaluated using repeated stratified sampling within the same cohort. While this internal validation approach helps mitigate overfitting, it does not constitute a true external validation. The generalizability of the SVM and logistic regression models to entirely new, independent populations of patients with schizophrenia and HCs remains to be demonstrated. Future studies with multicenter designs and independent validation cohorts are necessary to confirm the robustness and clinical transportability of these models. A second major limitation is the significant age difference between the schizophrenia and HCs groups, with HCs being notably younger. Although ANCOVA results indicated that age did not fully account for the observed group differences in VR metrics, age is closely linked to cognitive performance and familiarity with VR technology [37], which may have influenced our findings. In future studies, we will strictly recruit age-matched participants (±3 years) to eliminate this confounding factor and further validate the specificity of VR metrics for schizophrenia-related cognitive impairment. Third, the potential for learning effects with repeated use was not addressed in this cross-sectional design. While we omitted a practice session to minimize confounding effects on the initial assessment, repeated exposure to the “Fruit Pioneer” game in a longitudinal or periodic monitoring context could introduce performance improvements unrelated to cognitive change, thereby confounding the interpretation of scores over time. This is an important consideration for the tool’s application in long-term monitoring. Another limitation is the relatively small sample size of this study, which may affect the stability and generalizability of our ML models. Future multicenter studies with larger cohorts are needed to confirm our findings. Additionally, we did not collect longitudinal data, so the ability of VR metrics to track changes in cognitive function over time remains unclear.

### Conclusion

This study explored the application of the VR serious game “Fruit Pioneer” in the cognitive assessment of patients with stable schizophrenia. Methodologically, our study is innovative in its synergistic use of a purpose-built serious game for multidomain cognitive profiling and ML for pattern recognition and classification. This integrated approach differs from existing studies by not only demonstrating correlation with B-CATS but also providing a data-driven discriminative model. This work may contribute to the field by establishing a scalable digital assessment correlated with standard cognitive tests. Regarding real-world implications, the immersive nature of the VR serious game makes it a promising tool for practical clinical settings. It could serve as a potentially more acceptable method for routine cognitive screening and long-term monitoring in schizophrenia, thereby facilitating earlier identification of cognitive deficits and informing personalized rehabilitation strategies. Future research should further validate and optimize the ML models with larger, multicenter samples, explore the longitudinal use of the game for tracking cognitive change, and investigate its applicability across diverse clinical subgroups.

## Appendix

Multimedia Appendix 1 Supplementary materials.

## Abbreviations

AF: Animal Fluency
AUC: area under the curve
B-CATS: Brief Cognitive Assessment Tool for Schizophrenia
DSM-5: Diagnostic and Statistical Manual of Mental Disorders, Fifth Edition
DSST: Digital Symbol Substitution Test
GEQ-Core Module: Game Experience Questionnaire Core Module
HC: healthy control
LRM: logistic regression machine
ML: machine learning
PC: principal component
PCA: principal component analysis
SSQ: Simulator Sickness Questionnaire
SVM: support vector machine
TMTA: Trail Making Test Part A
TMTB: Trail Making Test Part B
VR: virtual reality

## Data Availability

The datasets generated or analyzed during this study are not publicly available due to the inclusion of sensitive participant information and the need to protect participant privacy but are available from the corresponding author on reasonable request.

## References

[1] Charlson FJ, Ferrari AJ, Santomauro DF, Diminic S, Stockings E, Scott JG, McGrath JJ, Whiteford HA (2018). Global epidemiology and burden of schizophrenia: findings from the Global Burden of Disease Study 2016. Schizophr Bull.

[2] Fioravanti M, Bianchi V, Cinti ME (2012). Cognitive deficits in schizophrenia: an updated metanalysis of the scientific evidence. BMC Psychiatry.

[3] Green MF (2016). Impact of cognitive and social cognitive impairment on functional outcomes in patients with schizophrenia. J Clin Psychiatry.

[4] Nuechterlein KH, Nasrallah H, Velligan D (2025). Measuring cognitive impairments associated with schizophrenia in clinical practice: overview of current challenges and future opportunities. Schizophr Bull.

[5] Martin-Key NA, Spadaro B, Funnell E, Barker EJ, Schei TS, Tomasik J, Bahn S (2022). The current state and validity of digital assessment tools for psychiatry: systematic review. JMIR Ment Health.

[6] Caporusso E, Melillo A, Perrottelli A, Giuliani L, Marzocchi FF, Pezzella P, Giordano GM (2025). Current limitations in technology-based cognitive assessment for severe mental illnesses: a focus on feasibility, reliability, and ecological validity. Front Behav Neurosci.

[7] Cuesta MJ, Pino O, Guilera G, Rojo JE, Gómez-Benito J, Purdon SE, Franco M, Martínez-Arán A, Segarra N, Tabarés-Seisdedos R, Vieta E, Bernardo M, Crespo-Facorro B, Mesa F, Rejas J (2011). Brief cognitive assessment instruments in schizophrenia and bipolar patients, and healthy control subjects: a comparison study between the Brief Cognitive Assessment Tool for Schizophrenia (B-CATS) and the Screen for Cognitive Impairment in Psychiatry (SCIP). Schizophr Res.

[8] Park MJ, Kim DJ, Lee U, Na EJ, Jeon HJ (2019). A literature overview of virtual reality (VR) in treatment of psychiatric disorders: recent advances and limitations. Front Psychiatry.

[9] Jin R, Pilozzi A, Huang X (2020). Current cognition tests, potential virtual reality applications, and serious games in cognitive assessment and non-pharmacological therapy for neurocognitive disorders. J Clin Med.

[10] Checa D, Bustillo A (2019). A review of immersive virtual reality serious games to enhance learning and training. Multimed Tools Appl.

[11] Cognolato M, Atzori M, Müller H (2018). Head-mounted eye gaze tracking devices: an overview of modern devices and recent advances. J Rehabil Assist Technol Eng.

[12] Mohammadi A, Hesami E, Kargar M, Shams J (2018). Detecting allocentric and egocentric navigation deficits in patients with schizophrenia and bipolar disorder using virtual reality. Neuropsychol Rehabil.

[13] Miskowiak KW, Jespersen AE, Kessing LV, Aggestrup AS, Glenthøj LB, Nordentoft M, Ott CV, Lumbye A (2022). Cognition assessment in virtual reality: validity and feasibility of a novel virtual reality test for real-life cognitive functions in mood disorders and psychosis spectrum disorders. J Psychiatr Res.

[14] Mitsea E, Drigas A, Skianis C (2025). A systematic review of serious games in the era of artificial intelligence, immersive technologies, the metaverse, and neurotechnologies: transformation through meta-skills training. Electronics.

[15] Tai AMY, Albuquerque A, Carmona NE, Subramanieapillai M, Cha DS, Sheko M, Lee Y, Mansur R, McIntyre RS (2019). Machine learning and big data: implications for disease modeling and therapeutic discovery in psychiatry. Artif Intell Med.

[16] Wang X, Yan C, Yang PY, Xia Z, Cai XL, Wang Y, Kwok SC, Chan RCK (2024). Unveiling the potential of machine learning in schizophrenia diagnosis: a meta-analytic study of task-based neuroimaging data. Psychiatry Clin Neurosci.

[17] Chen R, Greenwood TA, Braff Dl, Lazzeroni LC, Swerdlow NR, Calkins ME, Freedman R, Green MF, Gur RC, Gur RE, Nuechterlein KH, Radant AD, Silverman JM, Stone WS, Sugar CA, Tsuang MT, Turetsky BI, Light GA, Tsuang DW (2026). Machine learning enables efficient neurocognitive profiling in patients with schizophrenia. Nat Ment Health.

[18] Hudon A, Beaudoin M, Phraxayavong K, Potvin S, Dumais A (2023). Enhancing predictive power: integrating a linear support vector classifier with logistic regression for patient outcome prognosis in virtual reality therapy for treatment-resistant schizophrenia. J Pers Med.

[19] Zuo M, Chen X, Sui L (2025). Evaluation of machine learning algorithms for classification of visual stimulation-induced EEG signals in 2D and 3D VR videos. Brain Sci.

[20] Wang X, Kou X, Meng X, Yu J (2022). Effects of a virtual reality serious game training program on the cognitive function of people diagnosed with schizophrenia: a randomized controlled trial. Front Psychiatry.

[21] Hurford IM, Ventura J, Marder SR, Reise SP, Bilder RM (2018). A 10-minute measure of global cognition: validation of the Brief Cognitive Assessment Tool for Schizophrenia (B-CATS). Schizophr Res.

[22] Bouchard S, Berthiaume M, Robillard G, Forget H, Daudelin-Peltier C, Renaud P, Blais C, Fiset D (2021). Arguing in favor of revising the factor structure when assessing side effects induced by immersions in virtual reality. Front Psychiatry.

[23] Kennedy RS, Lane NE, Berbaum KS, Lilienthal MG (1993). Simulator Sickness Questionnaire: an enhanced method for quantifying simulator sickness. Int J Aviat Psychol.

[24] IJsselsteijn WA, de Kort YAW, Poels K (2013). The Game Experience Questionnaire.

[25] Poels K, de Kort YAW, IJsselsteijn WA (2007). D3.3: game experience questionnaire: development of a self-report measure to assess the psychological impact of digital games. Technische Universiteit Eindhoven.

[26] Hoonakker M, Doignon-Camus N, Bonnefond A (2017). Sustaining attention to simple visual tasks: a central deficit in schizophrenia? A systematic review. Ann N Y Acad Sci.

[27] Ludwig CJH, Gilchrist ID (2002). Stimulus-driven and goal-driven control over visual selection. J Exp Psychol Hum Percept Perform.

[28] Oberauer K (2019). Working memory and attention - a conceptual analysis and review. J Cogn.

[29] Proulx MJ (2010). Size matters: large objects capture attention in visual search. PLoS One.

[30] Diamond A (2020). Executive functions. Handb Clin Neurol.

[31] Bari A, Robbins TW (2013). Inhibition and impulsivity: behavioral and neural basis of response control. Prog Neurobiol.

[32] Gao L, Yang R, Fan HZ, Wang LL, Zhao YL, Tan SP, Xiao CL, Zhou SJ (2023). Correlation between aggressive behavior and impulsive and aggressive personality traits in stable patients with schizophrenia. Neuropsychiatr Dis Treat.

[33] Horan WP, Green MF, Kring AM, Nuechterlein KH (2006). Does anhedonia in schizophrenia reflect faulty memory for subjectively experienced emotions?. J Abnorm Psychol.

[34] Cohen AS, Najolia GM, Brown LA, Minor KS (2011). The state-trait disjunction of anhedonia in schizophrenia: potential affective, cognitive and social-based mechanisms. Clin Psychol Rev.

[35] Martin EA, Castro MK, Li LY, Urban EJ, Moore MM (2019). Emotional response in schizophrenia to the "36 questions that lead to love": predicted and experienced emotions regarding a live social interaction. PLoS One.

[36] Huang B, Li S, Sun B, Lyu H, Xu W, Jiao J, Pan F, Hu J, Chen J, Chen Y, Huang M, Xu Y (2021). Verification of using virtual reality to evaluate deficiencies in cognitive function among patients with schizophrenia in the remission stage: a cross-sectional study. BMC Psychiatry.

[37] Tan NC, Lim JE, Allen JC, Wong WT, Quah JHM, Muthulakshmi P, Teh TA, Lim SH, Malhotra R (2022). Age-related performance in using a fully immersive and automated virtual reality system to assess cognitive function. Front Psychol.

