# Supplementary material for: Cognitive Function Assessment Using a Virtual Reality Serious Game System in Patients With Stable Schizophrenia: Prospective Cohort Study
Source: JMIR Serious Games. 2026 May 8;14:e83001. doi: 10.2196/83001 (PMC13197741; doi:10.2196/83001)
Supplement: Multimedia Appendix 1 [file games_v14i1e83001_app1.pdf]

Table S1 Correlations between B-CATS Cognitive Scores and Virtual Reality (VR) Serious Game Performance in Stable Patients with Schizophrenia (SZs)

Variables	B-CATS			
	DSST	TMTAscore	TMTBscore	AF
Total Game Score	0.325 (p=0.044*)	-0.444 (p=0.005*)	-0.328 (p=0.041*)	0.047 (p=0.777)
Average Reaction Time for Hits	-0.189 (p=0.249)	0.373 (p=0.019*)	0.199 (p=0.224)	-0.096 (p=0.562)
Average Time from Gaze to Hit	-0.229 (p=0.162)	0.228 (p=0.163)	0.113 (p=0.494)	-0.006 (p=0.972)
Gaze Hit Rate	0.155 (p=0.347)	-0.038 (p=0.818)	-0.149 (p=0.364)	-0.044 (p=0.792)
Non-Gaze Hit Rate	-0.087 (p=0.597)	0.062 (p=0.706)	0.026 (p=0.877)	0.305 (p=0.059)
Shortest Time from Gaze to Hit	-0.376 (p=0.018*)	0.251 (p=0.123)	0.371 (p=0.020*)	-0.131 (p=0.427)
Longest Time from Gaze to Hit	-0.017 (p=0.918)	-0.026 (p=0.875)	-0.025 (p=0.878)	-0.211 (p=0.196)
Bomb Score	-0.114 (p=0.488)	0.139 (p=0.399)	0.069 (p=0.676)	0.105 (p=0.526)
continuous hits	0.354 (p=0.027*)	-0.411 (p=0.009*)	-0.288 (p=0.075)	0.027 (p=0.871)

note: Correlation analyses were performed using Spearman's rank correlation coefficient (r) reported.

Abbreviations: B-CATS, Brief Cognitive Assessment Tool for Schizophrenia; DSST, Digit Symbol Substitution Test; TMTAscore, Trail Making Test Part A score; TMTBscore, Trail Making Test Part B score; AF, Animal Fluency; VR, Virtual Reality; SZs, stable patients with schizophrenia. *P< 0.05, **P< 0.001.

Table S2 Correlations between B-CATS Cognitive Scores and Virtual Reality (VR) Serious Game Performance in Healthy Controls (HCs)

Variables	B-CATS			
	DSST	TMTAscore	TMTBscore	AF
Total Game Score	0.225 (p=0.074)	-0.149 (p=0.240)	-0.191 (p=0.130)	0.167 (p=0.186)
Average Reaction Time for Hits	-0.029 (p=0.818)	0.174 (p=0.168)	0.019 (p=0.884)	-0.069 (p=0.586)
Average Time from Gaze to Hit	-0.041 (p=0.746)	0.185 (p=0.143)	0.151 (p=0.234)	-0.188 (p=0.136)
Gaze Hit Rate	-0.057 (p=0.654)	-0.070 (p=0.581)	-0.219 (p=0.082)	0.111 (p=0.384)
Non-Gaze Hit Rate	0.039 (p=0.758)	-0.050 (p=0.694)	0.100 (p=0.430)	-0.097 (p=0.446)
Shortest Time from Gaze to Hit	0.204 (p=0.105)	-0.174 (p=0.170)	-0.013 (p=0.919)	0.074 (p=0.560)
Longest Time from Gaze to Hit	0.062 (p=0.625)	0.018 (p=0.887)	0.119 (p=0.349)	0.207 (p=0.101)
Bomb Score	-0.215 (p=0.087)	-0.021 (p=0.867)	0.073 (p=0.564)	-0.181 (p=0.153)
continuous hits	0.041 (p=0.750)	0.032 (p=0.803)	0.280 (p=0.025*)	0.041 (p=0.745)

note: Correlation analyses were performed using Spearman's rank correlation coefficient (r) reported.

Abbreviations: B-CATS, Brief Cognitive Assessment Tool for Schizophrenia; DSST, Digit Symbol Substitution Test; TMTAscore, Trail Making Test Part A score; TMTBscore, Trail Making Test Part B score; AF, Animal Fluency; VR, Virtual Reality; SZs, stable patients with schizophrenia. *P< 0.05, **P< 0.001

Table S3 Comparison of Game Experience Questionnaire (GEQ) Scores and Simulator Sickness Questionnaire (SSQ) Scores Between Stable Schizophrenia Patients (SZs) and Healthy Controls (HCs)

Variables	Dimensions	(M ± SD)/[M(Q1,Q3)]		t / Z	p
		HCs	SZs		
GEQ	Competence	11.78 ± 4.1	10.56 ± 4.79	1.319[b]	0.191
	Immersion	15 (12, 20)	14 (9.5, 19.5)	1395[a]	0.318
	Flow	11.73 ± 4.15	11 ± 5.22	0.746[b]	0.458
	Challenge	6.34 ± 3.19	6.74 ± 4.16	-0.515[b]	0.608
	Positive	15 (13, 18.25)	14 (8, 17)	1573.5[a]	0.026*
	Negative	1 (0, 2)	2 (1, 4)	912.5[a]	0.020*
	Tension	0 (0, 1.25)	1 (0, 3)	978.5[a]	0.045*
SSQ		2 (0, 5.25)	5 (1.5, 8.5)	972.5[a]	0.058

note: [a] Mann–Whitney U test; [b] t-test; *P< 0.05, **P< 0.001

Abbreviations: (M ± SD), (mean ± standard deviation); [M(Q1, Q3)], [median(first quartile, third quartile)]; GEQ: Game Experience Questionnaire Core Module; SSQ, Simulator Sickness Questionnaire; SZs, stable schizophrenia patients; HCs, healthy controls.

Table S4

Reseach Number: RZPGXM□□□□

Bief Cognitive Assessment Tool for Schizophenia (B-CATS)

Brief Cognitive Assessment Tool for Schizophrenia:

Construction of a Tool for Clinicians

Name: ....

Gender: ....

Age: ....

Registration Number: ....

Test Date: ....

Digit Symbol Substitution/Coding Test

⊃	≡	≻	×	Λ	=	*	∋	∞
1	2	3	4	5	6	7	8	9

⊃	Λ	≡	⊃	≻	=	≡	×	⊃	=		≡	⊃	=	⊃	≡

×	=	⊃	≡	Λ	=	≻	×	⊃	≡	=	∞	×	≻	∋

×	Λ	*	∋	⊃	≻	*	×	∋	Λ	≡	∞	≻	×	*

≡	×	Λ	⊃	=	×	⊃	Λ	=	*	∞	∋	≻	=	×

∞	Λ	∋	≻	=	*	×	Λ	≡	≻	*	∞	≡	∋	⊃

=	∞	*	≡	≻	=	×	∞	⊃	*	≡	Λ	=	∋	×

≡	∋	*	∞	≻	*	∋	Λ	⊃	∞	≡	⊃	×	≻	=

Λ	≡	⊃	=	×	≡	⊃	=	∞	*	≻	Λ	×	∋	∞

Score ______ （Number of correct responses within 90 seconds）

Trail Making Test (Part A)
Example

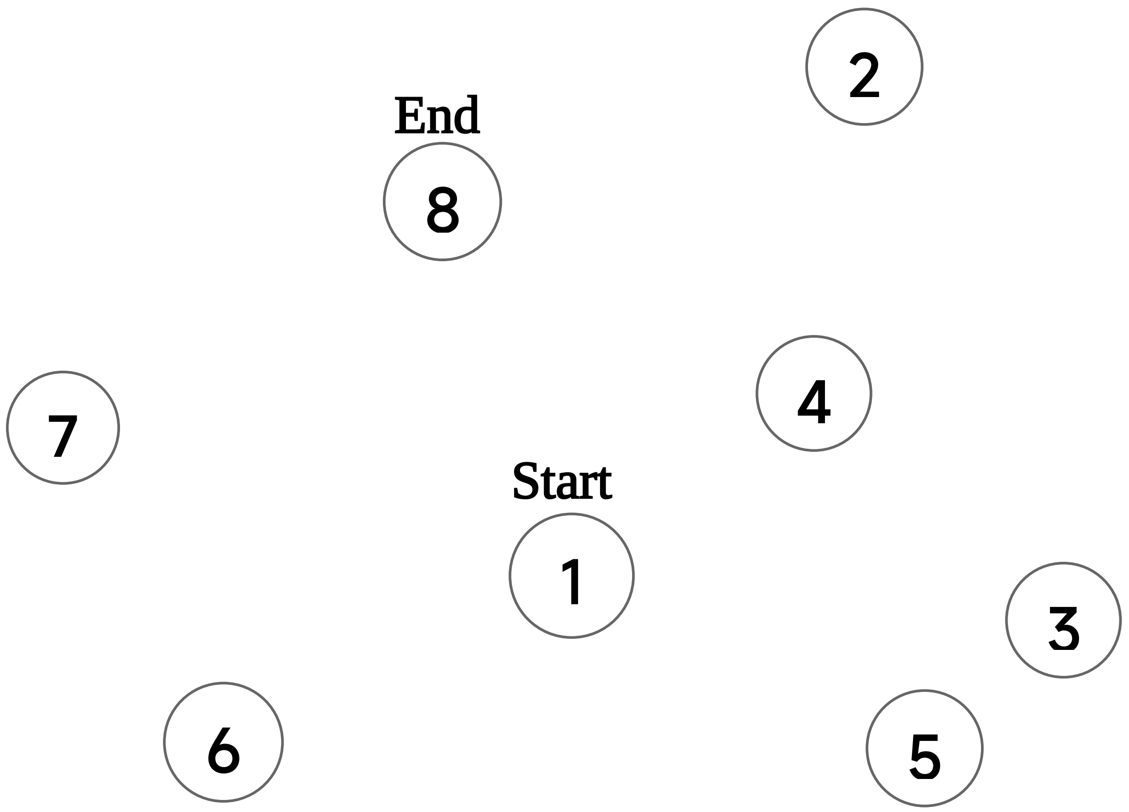

Trail Making Test (Part A)

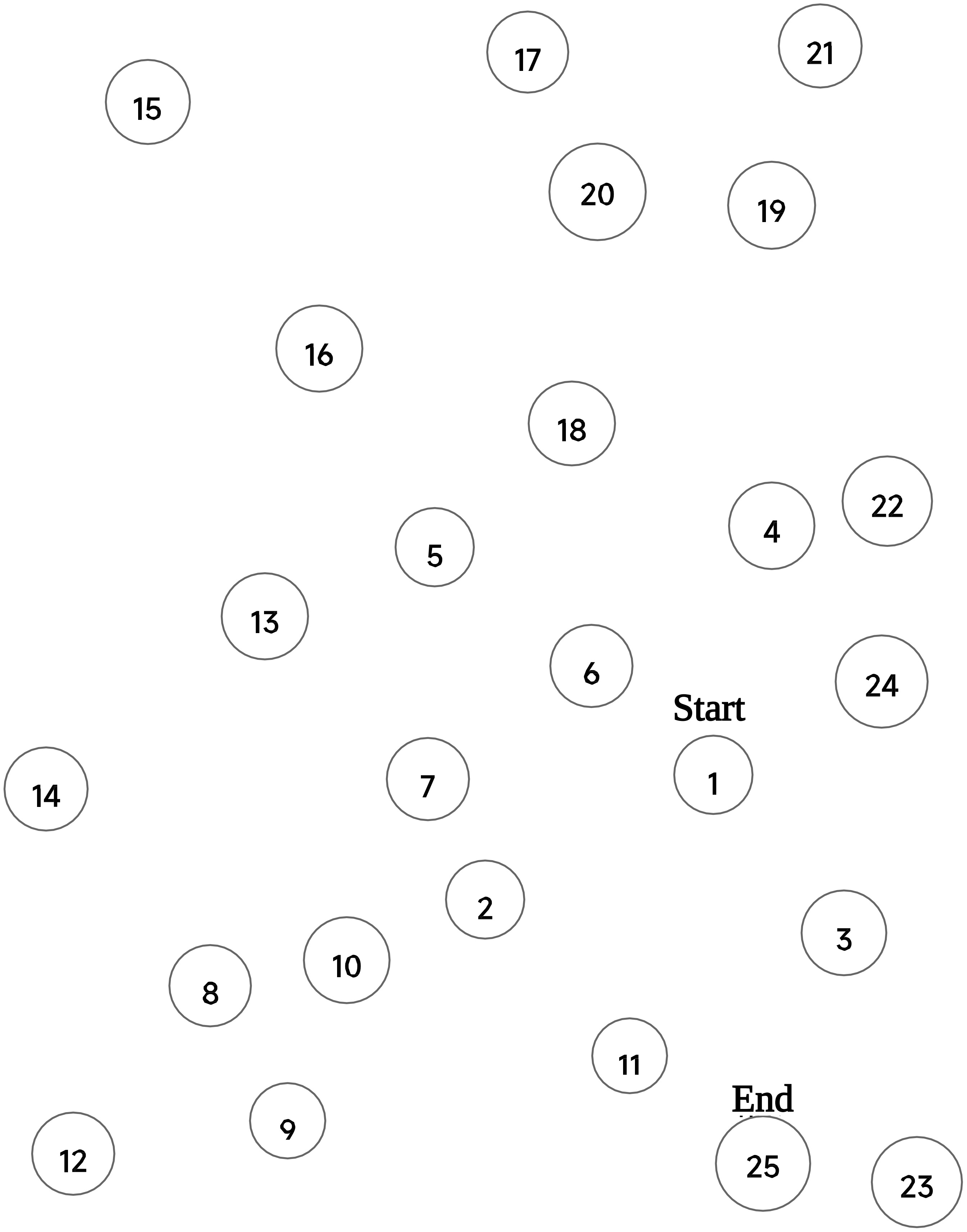

1. Total Completion time:___ seconds(0-300) (Recod300 if not completedwithin 300 seconds)
2. Number of Numerical Sequence Errors:____ seconds(0-40)
3. Number of Correct Lines: ____ seconds(0-24)

Trail Making Test (Part B)
Example

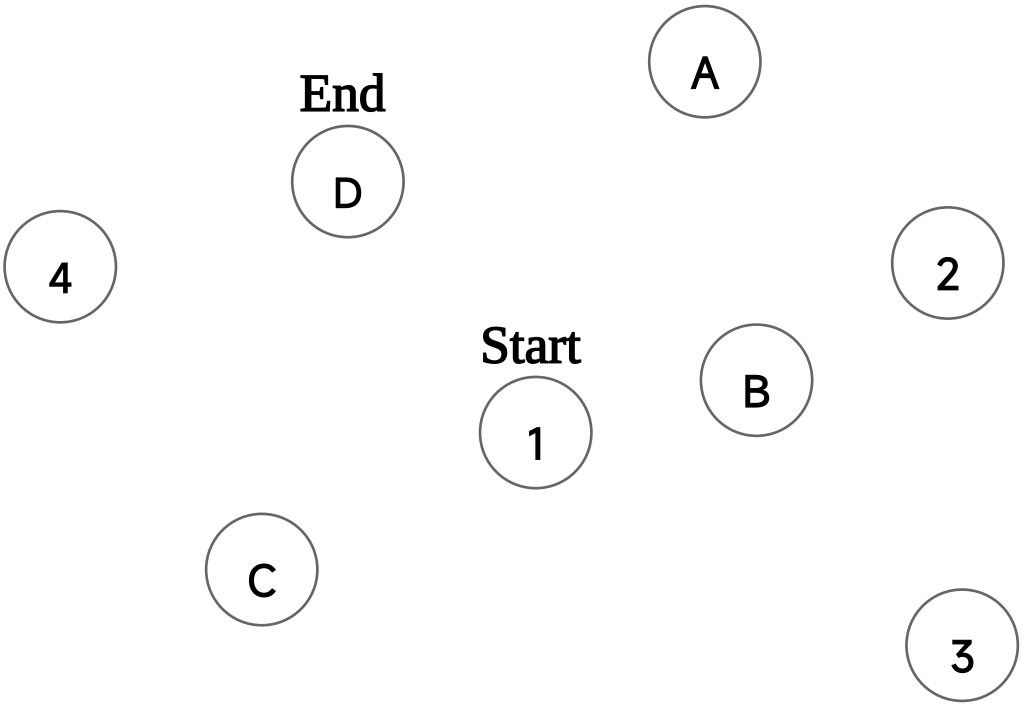

Trail Making Test (Part B)

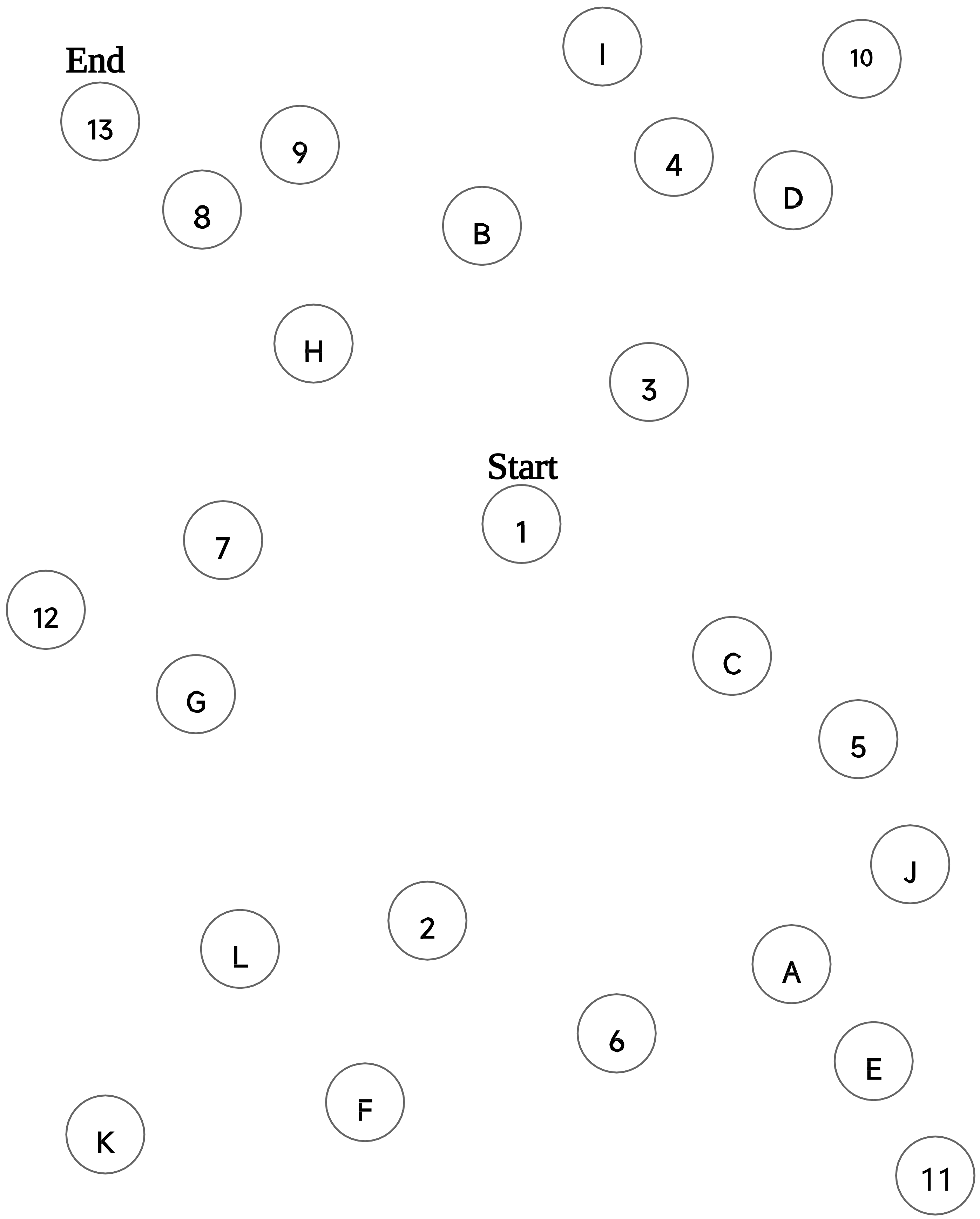

1. Total Completion time: ___ seconds(0-300) (Recod300 if not completedwithin 300 seconds)

2. Number of Numerical Sequence Errors: ____ seconds(0-40)

3. Number of Correct Lines: ____ seconds(0-24)

Semantic Fluency/Animal Naming Test (Time Limit: 60 seconds)

Please name as many animal species as possible, as quickly as you can. Any animal is acceptable—they can be from farms, jungles, oceans, or domestic pets. For example, you could say 'dog'. You will have one minute. Are you ready? Start.

1.	11.	21.	31.
2.	12.	22.	32.
3.	13.	23.	33.
4.	14.	24.	34.
5.	15.	25.	35.
6.	16.	26.	36.
7.	17.	27.	37.
8.	18.	28.	38.
9.	19.	29.	39.
10.	20.	30.	40.

Score________(Number of unique animals named)

Brief Assessment of Cognition in Schizophrenia (B-CATS) Scoring Rules

1. Digit Symbol Substitution Test (DSST)

Task Description: Participants are provided with a sheet containing a key (digits 1– 9 paired with unique symbols) at the top, followed by rows of symbols.

Time Limit: 120 seconds.

Scoring Criterion: Score is the total number of correct digit-symbol matches. The first 10 symbols on the sheet are examples and not scored; the maximum possible score is 110.

Assessed Domains: Processing speed, attentional control, visual working memory.

2. Trail Making Test Part A (TMTA)

Task Description: Participants draw a continuous line to connect numbered circles (1– 25) in ascending order without lifting the pen.

Error Correction: If an error occurs, participants are redirected to the last correct position to continue.

Scoring Criterion: Score is the total time (in seconds) to complete the task. A maximum of 300 seconds is recorded if the task is not finished within that timeframe.

Assessed Domains: Visual scanning speed, sustained attention, psychomotor processing speed.

3. Trail Making Test Part B (TMTB)

Task Description: Participants draw a continuous line to alternately connect numbered and lettered circles (1-A-2-B-...-13) in ascending alphanumeric order without lifting the pen.

Error Correction: If an error occurs, participants are redirected to the last correct position to continue.

Scoring Criterion: Score is the total time (in seconds) to complete the task. A maximum of 300 seconds is recorded if the task is not finished within that timeframe.

Assessed Domains: Executive function, task-switching ability, inhibitory control.

4. Animal Fluency (AF)

Task Description: Participants verbally list as many unique animal names as possible.

Time Limit: 60 seconds.

Scoring Criterion: Score is the total number of valid, distinct animal names reported. Duplicate names or non-animal terms are not counted.

Assessed Domains: Semantic memory, lexical access efficiency, strategic retrieval processes.

References：Hurford I M, Ventura J, Marder S R, et al. A 10-minute measure of global cognition: Validation of the Brief Cognitive Assessment Tool for Schizophrenia (B-CATS)[J]. Schizophrenia research, 2018, 195: 327-333.

研究编号：RZPGXM□□□□

B-CATS 认知评估量表

Brief Cognitive Assessment Tool for Schizophrenia:

Construction of a Tool for Clinicians

姓　　名 ：..

性　　别 ：..

年　　龄 ：..

登 记 号 ：..

测试日期 ：..

治疗药物 ：

数字符号替换/编码测验

⊃	≡	>	×	Λ	=	*	∋	∞
1	2	3	4	5	6	7	8	9

⊃	∧	≡	⊃	>	=	≡	×	⊃	=		≡	⊃	=	⊃	≡

×	=	⊃	≡	∧	=	>	×	⊃	≡	=	∞	×	>	∋

×	∧	*	∋	⊃	>	*	×	∋	∧	≡	∞	>	×	*

≡	×	∧	⊃	=	×	⊃	Λ	=	*	∞	∋	>	=	×

∞	∧	∋	>	=	*	×	∧	≡	>	*	∞	≡	∋	⊃

=	∞	*	≡	>	=	×	∞	⊃	*	≡	∧	=	∋	×

≡	∋	*	∞	>	*	∋	∧	⊃	∞	≡	⊃	×	>	=

∧	≡	⊃	=	×	≡	⊃	=	∞	*	>	∧	×	∋	∞

得分 ________________（90 秒内的正确数）

连线测试（A 部分）

样例

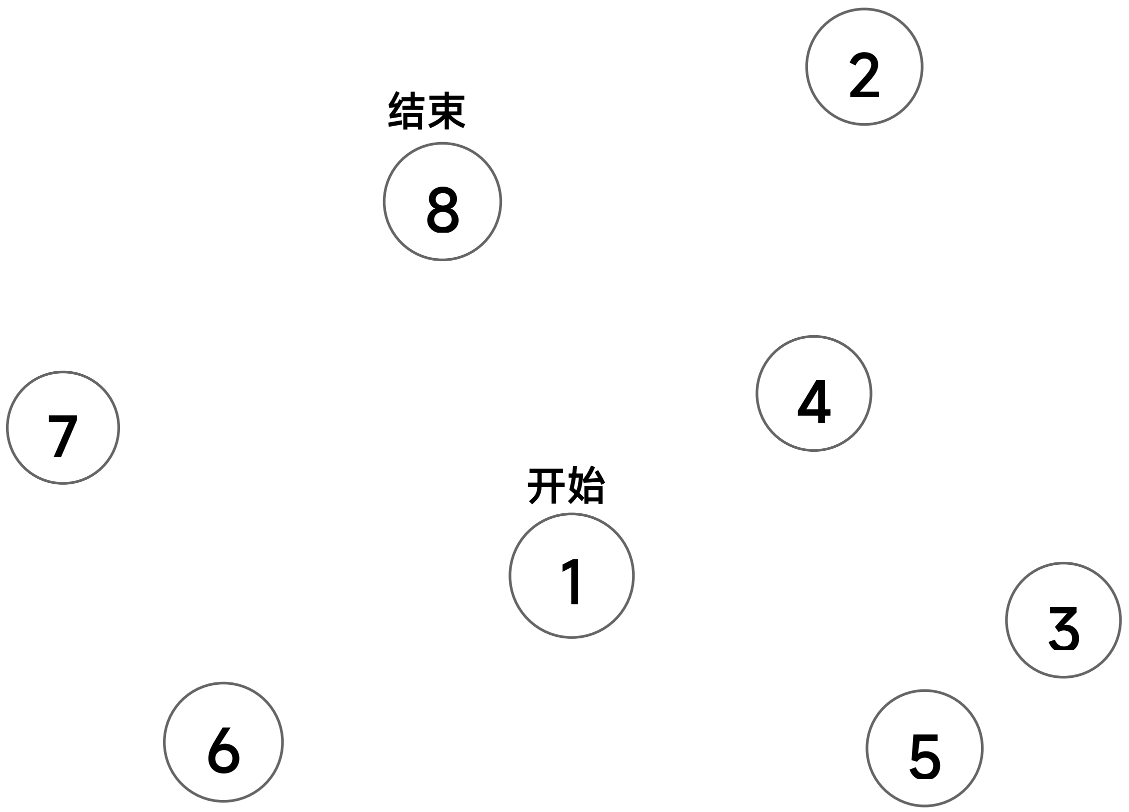

连线测验（A部分）·测试页

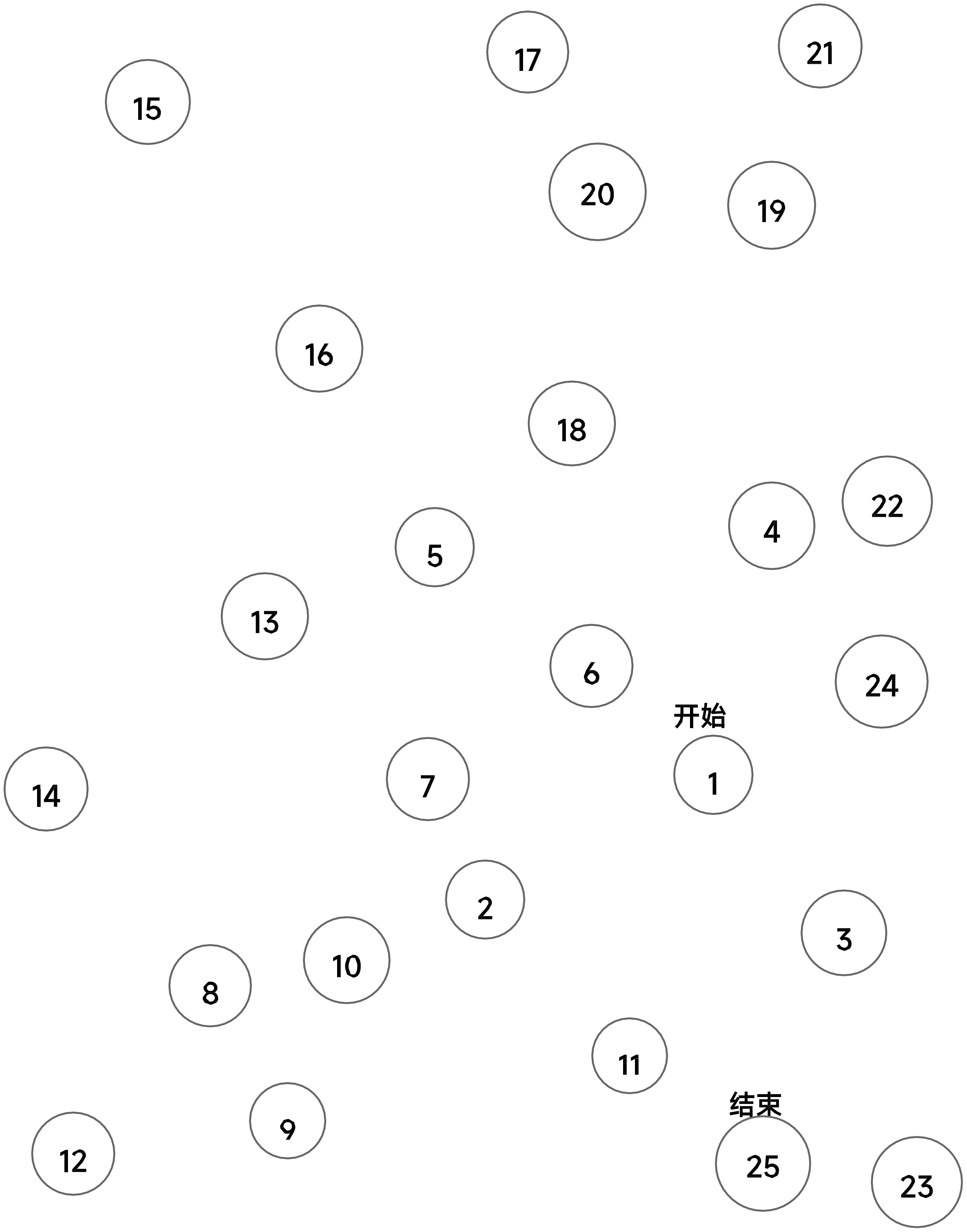

1. **全部数字完成时间 __________ （0~300）秒** （如超出300秒未完成，记录300）
2. **数字顺序错误数 ____________ （0~40）**
3. **正确的线条数 _______________ （0~24）**

连线测试（B 部分）

样例

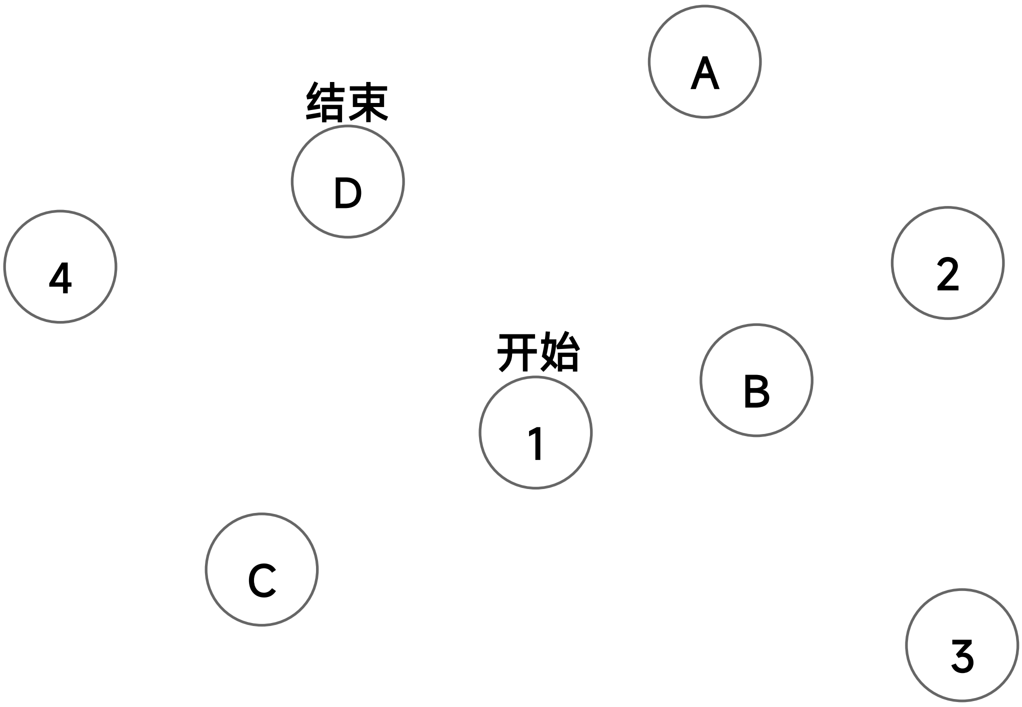

连线部分（B 部分）·测试页

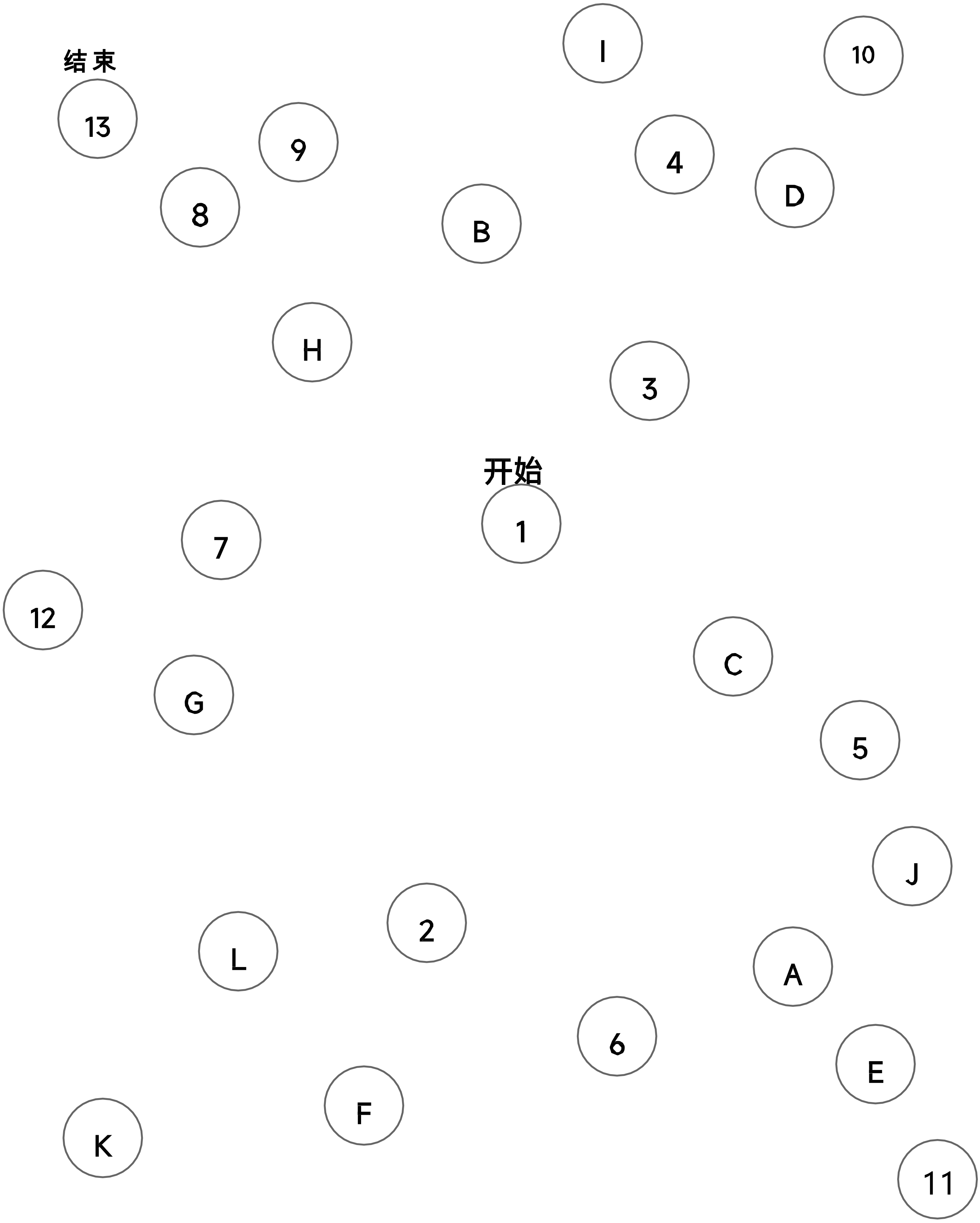

1. **全部数字完成时间 __________ （0~300）秒** （如超出300秒未完成，记录300）
2. **数字顺序错误数 ___________ （0~40）**
3. **正确的线条数 ______________ （0~24）**

语义流畅性/动物命名测验（限时 60 秒）

现在请尽可能多的告诉我你能记得的动物名称，尽可能快的说，任何动物都可以：它们可以来自农场、丛林、海洋或家畜。例如，你可以说狗。给你一分钟时间，尽可能说的多。准备好了吗？开始。

1.	11.	21.	31.
2.	12.	22.	32.
3.	13.	23.	33.
4.	14.	24.	34.
5.	15.	25.	35.
6.	16.	26.	36.
7.	17.	27.	37.
8.	18.	28.	38.
9.	19.	29.	39.
10.	20.	30.	40.

得分_______________________

精神分裂症简明认知评估工具（B-CATS）计分规则

1. 数字符号替换测验（DSST）
任务描述：测试表顶部提供数字 1-9 与对应独特符号的配对密钥，下方为多行数字，每个数字下方有空白方框。
时间限制：120 秒。
计分标准：得分 = 正确完成的数字 - 符号配对数量。测试表共含 120 个符号，前 10 个为示例不计分，最高得分 110 分。
评估维度：加工速度、注意力控制、视觉工作记忆。
2. 连线测验 A 部分（TMTA）
任务描述：测试表上有散落的带数字圆圈（1-25），被试需在不抬笔的情况下，按升序连续绘制线条连接数字。
错误修正：若出现连接错误，需引导被试回到最后正确位置后继续完成。
计分标准：得分 = 完成任务的总时间（秒）。300 秒内未完成则记录为 300 秒。
评估维度：视觉扫描速度、持续注意力、心理运动加工速度。
3. 连线测验 B 部分（TMTB）
任务描述：测试表上有散落的带数字或字母的圆圈，被试需在不抬笔的情况下，按字母数字升序交替绘制线条连接（1-A-2-B-…-13）。
错误修正：若出现连接错误，需引导被试回到最后正确位置后继续完成。
计分标准：得分 = 完成任务的总时间（秒）。300 秒内未完成则记录为 300 秒。
评估维度：执行功能、任务转换能力、抑制控制（需抑制固有的数字排序倾向）。
4. 动物流畅性测验（AF）
任务描述：被试需在规定时间内，口头列举尽可能多的独特动物名称。
时间限制：60 秒。
计分标准：得分 = 有效且独特的动物名称总数。重复名称或非动物类词汇不计分。
评估维度：语义记忆（依赖前颞叶介导的类别知识提取）、词汇提取效率（依赖左额下回支持的快速词汇检索）、策略性提取过程（依赖背外侧前额叶皮层参与的执行引导聚类 / 转换策略），反映额颞叶网络整合功能及执行控制作用。

References：Hurford I M, Ventura J, Marder S R, et al. A 10-minute measure of global cognition: Validation of the Brief Cognitive Assessment Tool for Schizophrenia (B-CATS)[J]. Schizophrenia research, 2018, 195: 327-333.

Table S5

Simulator Sickness Questionnaire (SSQ)

Item	Content	None	Mild	Moderate	Severe
1	Do you feel general discomfort?	0	1	2	3
2	Do you feel tired?	0	1	2	3
3	Do you have a headache?	0	1	2	3
4	Do you feel eye strain?	0	1	2	3
5	Do you have difficulty focusing on something?	0	1	2	3
6	Do you experience increased saliva production?	0	1	2	3
7	Are you sweating?	0	1	2	3
8	Do you feel nauseous?	0	1	2	3
9	Do you currently have difficulty concentrating?	0	1	2	3
10	Do you feel a swollen head?	0	1	2	3
11	Do you have blurred vision?	0	1	2	3
12	Do you feel dizzy (when eyes are open)?	0	1	2	3
13	Do you feel dizzy (when eyes are closed)?	0	1	2	3
14	Do you feel vertigo?	0	1	2	3
15	Do you feel abnormal sensations in your stomach?	0	1	2	3
16	Are you burping currently?	0	1	2	3

Core Scoring Rules

Scoring Basis: A 4-point Likert scale is adopted, with scores assigned according to symptom severity: None (0 points), Mild (1 point), Moderate (2 points), and Severe (3 points).

Total Score Calculation: An unweighted scoring method is used. The total score is obtained by summing the scores of all 16 items directly, without applying the traditional weighted formula (to avoid inflating the total score, which aligns with the revised recommendations for the scale in VR scenarios). A higher total score indicates more severe discomfort induced by VR immersion.

Reference: Bouchard S, Berthiaume M, Robillard G, et al. Arguing in favor of revising the simulator sickness questionnaire factor structure when assessing side effects induced by immersions in virtual reality[J]. Frontiers in Psychiatry, 2021, 12: 73974

模拟器不适感量表,SSQ

序号	条目	无 ——→ 严重			
1	您是否有一般不适的感觉?	0	1	2	3
2	您是否感到疲倦?	0	1	2	3
3	您是否感到头痛?	0	1	2	3
4	您是否感到眼睛疲劳?	0	1	2	3
5	您是否感到难以聚焦某处?	0	1	2	3
6	您是否感到口水分泌增加?	0	1	2	3
7	您是否正在出汗?	0	1	2	3
8	您是否感到恶心?	0	1	2	3
9	您目前是否感到难以集中注意力?	0	1	2	3
10	您是否感到头胀?	0	1	2	3
11	您是否感到视线模糊?	0	1	2	3
12	您是否感到眼花(睁眼时)?	0	1	2	3
13	您是否感到眼花(闭眼时)?	0	1	2	3
14	您是否感到眩晕?	0	1	2	3
15	您是否感到胃部感觉异样?	0	1	2	3
16	您目前是否打嗝?	0	1	2	3

核心评分原则
计分基础：采用 4 级计分制，各条目按症状严重程度赋值：无（0 分）、轻微（1 分）、中度（2 分）、严重（3 分）。
总分计算：采用未加权计分法，直接将 16 个条目的得分求和，无需按传统公式加权（避免总分膨胀，符合 VR 场景下的量表修订建议）。得分越高，表明 VR 沉浸引发的不适感越严重。

Reference: Bouchard S, Berthiaume M, Robillard G, et al. Arguing in favor of revising the simulator sickness questionnaire factor structure when assessing side effects induced by immersions in virtual reality[J]. Frontiers in Psychiatry, 2021, 12: 73974

Table S6

Game Experience Questionnaire - Core Scale

Item	Content	Not at All	Slightly	Moderately	Highly	Extremely
1	I feel satisfied	0	1	2	3	4
2	I am skilled	0	1	2	3	4
3	I am interested in the game content	0	1	2	3	4
4	I think this game is fun	0	1	2	3	4
5	I am fully engaged in the game	0	1	2	3	4
6	I feel pleasant	0	1	2	3	4
7	This game makes me feel displeased	0	1	2	3	4
8	I think about other things while playing the game	0	1	2	3	4
9	Playing this game makes me feel tired	0	1	2	3	4
10	I can play this game well	0	1	2	3	4
11	I think this game is difficult	0	1	2	3	4
12	I think this game is visually enjoyable	0	1	2	3	4
13	I forget everything happening around me	0	1	2	3	4
14	I feel good	0	1	2	3	4
15	I perform well in this game	0	1	2	3	4
16	I feel bored	0	1	2	3	4
17	I feel a sense of accomplishment	0	1	2	3	4
18	This game enriches my imagination	0	1	2	3	4
19	I feel like I have explored new things	0	1	2	3	4
20	I enjoy it	0	1	2	3	4
21	I can quickly achieve the goals in the game	0	1	2	3	4
22	I feel annoyed	0	1	2	3	4
23	I feel stressed	0	1	2	3	4
24	I feel anxious	0	1	2	3	4
25	I lose track of time	0	1	2	3	4
26	I think the game is challenging	0	1	2	3	4
27	I am deeply attracted to the game	0	1	2	3	4
28	I am very focused on the game	0	1	2	3	4
29	I feel frustrated	0	1	2	3	4
30	I feel playing this game is a rich experience	0	1	2	3	4
31	I feel disconnected from the outside world	0	1	2	3	4
32	I feel time is pressing	0	1	2	3	4
33	I feel I have put in great effort	0	1	2	3	4

Core Scoring Rules

This study adopted the Game Experience Questionnaire Core Module (GEQ-Core Module) designed by IJsselsteijn et al. The questionnaire covers essential content necessary for assessing gaming experience, consisting of 7 dimensions with a total of 33 items. The 7 dimensions are: Competence, Sensory and Imaginative Immersion, Flow, Challenge, Positive Affect, Negative Affect, and Tension/Annoyance.

The items included in each dimension are as follows:

Competence: Items 2, 10, 15, 17, 21

Sensory and Imaginative Immersion: Items 3, 12, 18, 19, 27, 30

Flow: Items 5, 13, 25, 28, 31

Challenge: Items 11, 23, 26, 32, 33

Positive Affect: Items 1, 4, 6, 14, 20

Negative Affect: Items 7, 8, 9, 16

Tension/Annoyance: Items 22, 24, 29

A 5-point Likert scale was used for scoring: 0 = Not at All, 1 = Slightly, 2 = Moderately, 3 = Highly, 4 = Extremely. The average score of each dimension was calculated to evaluate differences in participants' gaming experiences across various dimensions.

References：IJsselsteijn W A, De Kort Y A W, Poels K. The game experience questionnaire[J]. 2013.1

游戏体验问卷—核心量表

条目	内容	根本不	有一点	中度	重度	极度
1	我感到满意	0	1	2	3	4
2	我是有技巧的	0	1	2	3	4
3	我对这个游戏的内容感兴趣	0	1	2	3	4
4	我认为这个游戏是有趣的	0	1	2	3	4
5	我完全投入在游戏中	0	1	2	3	4
6	我感到愉快	0	1	2	3	4
7	这个游戏让我感到不悦	0	1	2	3	4
8	玩这个游戏的时候我在想其他事	0	1	2	3	4
9	玩这个游戏让我感到疲惫	0	1	2	3	4
10	我能玩好这个游戏	0	1	2	3	4
11	我认为这个游戏很难	0	1	2	3	4
12	我认为这个游戏是视觉上的享受	0	1	2	3	4
13	我忘记了周围发生的所有事	0	1	2	3	4
14	我感觉良好	0	1	2	3	4
15	我在这个游戏上玩得很好	0	1	2	3	4
16	我感到无聊	0	1	2	3	4
17	我感到有成就感	0	1	2	3	4
18	我感到游戏丰富了我的想象力	0	1	2	3	4
19	我感觉探索了新事物	0	1	2	3	4
20	我享受其中	0	1	2	3	4
21	我能很快达到游戏中的目标	0	1	2	3	4
22	我感到烦恼	0	1	2	3	4
23	我感到有压力	0	1	2	3	4
24	我感到焦躁	0	1	2	3	4
25	我忘记了时间的存在	0	1	2	3	4
26	我觉得游戏具有挑战性	0	1	2	3	4
27	我被游戏深深吸引	0	1	2	3	4
28	我非常专注于游戏	0	1	2	3	4
29	我感到沮丧	0	1	2	3	4
30	我感觉玩这个游戏是段丰富的体验	0	1	2	3	4
31	我感觉失去了与外界的联系	0	1	2	3	4
32	我感到时间紧迫	0	1	2	3	4
33	我感到付出了很大努力	0	1	2	3	4

评分规则

本研究采用由艾塞尔施泰因（IJsselsteijn）等人设计的游戏体验问卷 - 核心量表（Game Experience Questionnaire Core Module, GEQ-Core Module）。

该问卷涵盖了游戏体验评估所需的核心内容，共包含 7 个维度、33 个条目。7 个维度分别为：能力感（Competence）、感官与想象沉浸感（Sensory and Imaginative Immersion）、流畅感（Flow）、挑战性（Challenge）、积极情感（Positive Affect）、消极情感（Negative Affect）、紧张 / 烦恼感（Tension/Annoyance）。

各维度包含的条目如下：

能力感（Competence）：条目 2、10、15、17、21

感官与想象沉浸感（Sensory and Imaginative Immersion）：条目 3、12、18、19、27、30

流畅感（Flow）：条目 5、13、25、28、31

挑战性（Challenge）：条目 11、23、26、32、33

积极情感（Positive Affect）：条目 1、4、6、14、20

消极情感（Negative Affect）：条目 7、8、9、16

紧张 / 烦恼感（Tension/Annoyance）：条目 22、24、29

问卷采用 5 级李克特（Likert）评分法：0 分 = 根本不，1 分 = 有一点，2 分 = 中度，3 分 = 重度，4 分 = 极度。通过计算各维度的平均得分，评估参与者在不同维度上的游戏体验差异。

References：IJsselsteijn W A, De Kort Y A W, Poels K. The game experience questionnaire[J]. 2013.**1**

Figure S1

A

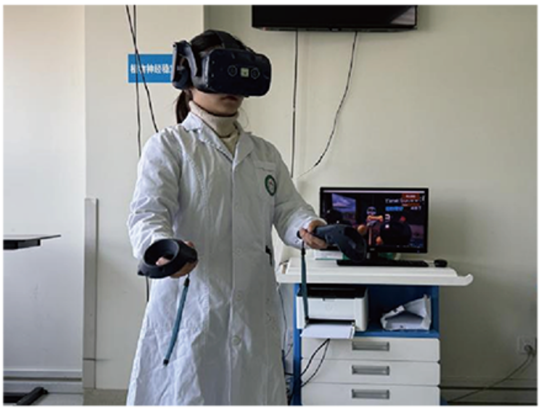

B

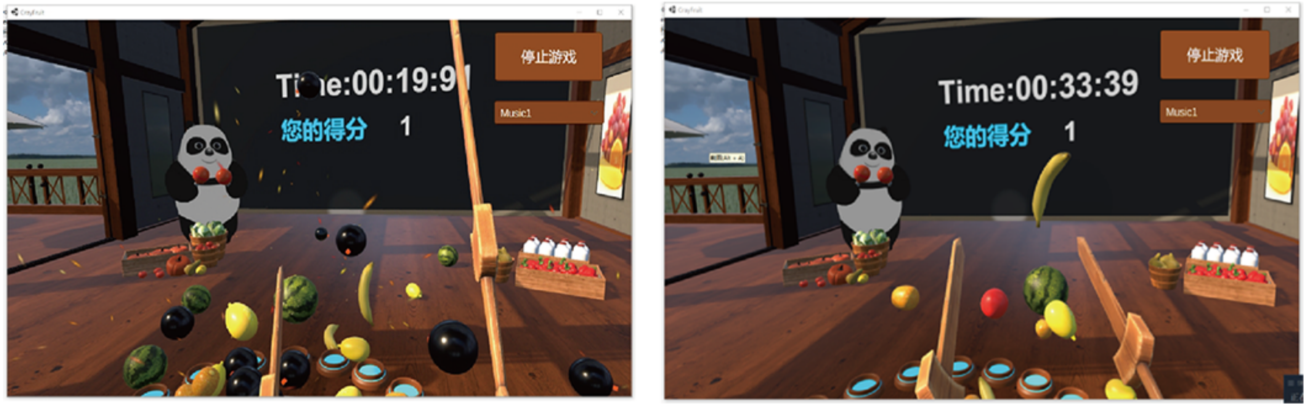

Figure S1 A: A research team member (medical staff) demonstrating the use of VR equipment for the "Fruit Pioneer" cognitive assessment.

Note: To protect patient privacy, this image features a study team member, not a study participant, to illustrate the standard procedure for operating the VR device during assessment.

Figure S1 B: Screenshot of the virtual reality (VR) serious game Fruit Pioneer used for cognitive function assessment

This screenshot displays the first-person perspective of the custom-designed VR fruit-slicing game, with haptic controllers rendered as 2 long swords in the foreground. 5 types of edible fruits (banana, apple, watermelon, orange, and pineapple) and black spherical bombs are launched from multiple nozzles at the base of the play area; participants earn points by slicing the 5 fruits and incur score deductions for slicing the black spherical bombs. A cartoon panda character is visible in the background, and a real-time scoreboard in the upper interface shows the participant's current game score and elapsed play time.
